# Perceived Healthcare Service Quality of Patients in the Post COVID‐19 Pandemic: A Cross‐Sectional Study at a Public District Hospital in Vietnam

**DOI:** 10.1002/hsr2.73018

**Published:** 2026-08-10

**Authors:** Nguyen Duc Thanh, Tran Chi Thanh, Ha Thi Minh Nguyet, Chu Huyen Xiem

**Affiliations:** ^1^ Health Management Training Institute Hanoi University of Public Health Dong Ngac Commune Hanoi Vietnam; ^2^ University of Medicine and Pharmacy at Ho Chi Minh city Ho Chi Minh City Vietnam

**Keywords:** healthcare service quality, outpatients, post COVID‐19 pandemic, SERVPERF, Vietnam

## Abstract

**Background and Aim:**

After the COVID‐19 pandemic, Vietnamese hospitals experienced reduced patient numbers, staffing shortages, and decreased morale, adversely affecting service quality and patient satisfaction. This study aimed to assess patients' perceptions of healthcare service quality at a primary facility in the post‐pandemic context and compare these findings with pre‐pandemic benchmarks.

**Methods:**

A cross‐sectional study conducted at a public district hospital surveyed outpatient clients between November 1, 2022 and June 30, 2023 using the SERVPERF tool for quality assessment. Data were analyzed using descriptive statistics and bivariate analysis.

**Results:**

A total of 190 outpatients participated (58.9%, *n* = 112 female; majority aged 18–44; 83.7% (*n* = 159) with high school education or below). The tool showed good reliability (Cronbach's alpha of 0.96), with 75.3% (*n* = 143) reporting “Good” perceived healthcare service quality (PHSQ). Factors influencing PHSQ included age, income, and education level.

**Conclusion:**

This study provides a post‐pandemic assessment of PHSQ, showing high SERVPERF scores but a relatively low proportion of “good” PHSQ when compared to pre‐pandemic benchmarks.

## 1 Introduction

Hospitals, integral components of the healthcare system, offer a wide array of health services to the community, including medical services, medical support services, rehabilitation, and integrated health services [1]. Following the global COVID‐19 pandemic—which was declared a Public Health Emergency of International Concern by the WHO from March 2020 until May 2023 has resulted in a global death toll exceeding six million individuals [2]. Concomitant with the elevated mortality rates, there has been substantial disruption to healthcare services. The Vietnamese healthcare system entered a critical recovery phase. While the formal emergency status spanned over 3 years, the most significant disruptions to hospital operations in Vietnam occurred between 2020 and late 2021, leading to the shifts in patient volume addressed in this study. The Vietnamese Ministry of Health and independent researchers have noted significant shifts in healthcare utilization. While Vietnam was praised for its early containment strategies, the systemic impact of the pandemic led to a recorded decline in routine hospital admissions [3]. By 2023, while clinical cases of COVID‐19 were controlled, the “aftermath” period revealed that many public hospitals continued to face challenges in returning to pre‐pandemic patient volumes [4]. The pandemic acted as a “poverty trap,” reducing household income and increasing the poverty rate across Vietnam. This economic strain forced many to deprioritize or postpone medical care due to an inability to pay [4, 5]. There has been a significant trend toward the use of smartphone healthcare applications and telemedicine, as patients look for more convenient and safer ways to consult with providers [6]. Systemic vulnerabilities, such as shortages in medicines and medical supplies reported in 2022 and 2023, have contributed to patient dissatisfaction and a potential shift in trust toward facility‐based care [4].

In the aftermath of the COVID‐19 pandemic in Vietnam, the economy has plunged into a severe crisis, leading to financial strains across various sectors, including healthcare services. Compounding this situation are ongoing challenges such as the constrained service capacity of primary medical facilities at the grassroots level, particularly commune health stations, attributable to deficiencies in human resources, limited professional expertise among healthcare personnel, inadequate medical infrastructure and resources, as well as scarcity of essential medicines, medical equipment, and supplies [7]. The public health sector in Vietnam faced a severe shortage of qualified staff in the aftermath of the COVID‐19 pandemic, paralleling the widespread global shortage of healthcare workers caused by the pandemic. The healthcare worker job motivation in the post‐COVID‐19 pandemic was low, with a decline across all dimensions, as evidenced by a mean score of 3.26 (on a 5‐point Likert scale). Specifically, job satisfaction and organizational commitment experienced the most reported negative impact, with respective scores of 3.02 and 3.00 [8]. Consequently, a significant number of healthcare professionals resigned in various countries, indicating an unprecedented trend. One of the prominent causal factors identified for this phenomenon was employee attrition, primarily associated with the work environment including workload and occupational hazards exacerbated by the pandemic [9]. Furthermore, diminished staff motivation represents a significant determinant of substandard service quality within healthcare establishments, demonstrating a propensity towards staff impatience towards clientele, elevated absentee rates, prolonged wait intervals, unregulated fee impositions, and heightened incidence of labor‐related strikes [10, 11, 12, 13].

The pursuit of defining “healthcare service quality in the post‐COVID‐19 pandemic” remains an ongoing challenge, necessitating the rationale for conducting healthcare service quality research in the post‐COVID‐19 pandemic. Primarily, the pandemic has prompted a reevaluation and innovative approach to quality in the post‐COVID‐19 pandemic. There is acknowledgment of the potential harm to “normal” quality and safety activities. The identified “potential harm” pertains to the heightened strain on healthcare workers, still grappling with the repercussions of the COVID‐19 surge, engendered by the implementation of “normal quality and safety activities,” which encompass standard protocols and procedures that may further burden an already stressed and overworked healthcare workforce [14]. Additionally, during the COVID‐19 pandemic, public perception of healthcare service quality was adversely affected for several reasons. Healthcare systems became overwhelmed, leading to delays in non‐COVID‐19 care and frustration among patients. Access to care was disrupted due to lockdowns and fears of contracting the virus, contributing to a perception of reduced healthcare service quality. Concerns about safety and infection control measures within healthcare settings also impacted people's trust in the healthcare system. Communication challenges and the psychological impact of the pandemic further influenced negative perceptions of healthcare service quality [15]. Prior research in Vietnam using the SERVPERF tool consistently reported high levels of patient perception, with mean scores often exceeding 4.2 out of 5.0 in various provincial and district hospitals [16, 17, 18]. However, these studies were largely conducted under stable healthcare conditions. The COVID‐19 pandemic introduced significant systemic stressors, including staffing shortages and reduced medical supplies, which have been documented to affect healthcare delivery globally. In Vietnam, while preliminary reports suggested a decline in trust during the crisis, there is a lack of evidence regarding whether outpatient perceptions have stabilized or shifted in the long‐term aftermath (2023). To theoretically frame our evaluation of outpatient healthcare service quality, we utilized the SERVPERF (Service Performance) model. First proposed by Cronin and Taylor [19], this framework posits that measuring service quality based on perceived performance alone—rather than the “expectations‐minus‐perceptions” gap used in other models—provides a more accurate and robust prediction of overall service quality. The SERVPERF model assesses quality through five distinct dimensions:

- Tangibles: The appearance of physical facilities, equipment, and personnel.
- Reliability: The ability to perform the promised service dependably and accurately.
- Responsiveness: The willingness to help patients and provide prompt service.
- Assurance: The knowledge and courtesy of employees and their ability to inspire trust and confidence.
- Empathy: The provision of caring, individualized attention to patients.

This multidimensional framework has been successfully adapted and validated for use in public hospital settings, including the work by Rasyida et al. (2016), which demonstrated the tool's efficacy in capturing the nuances of patient‐provider interactions [2]. By adopting this validated framework, this study addresses this gap by assessing PHSQ at a public district hospital during the recovery phase to determine if previous quality benchmarks are still being met.

## 2 Methods

### 2.1 Study Design

A cross‐sectional investigation was conducted to assess the perception of the quality of healthcare services and the associated factors.

### 2.2 Research Duration and Location

The study was conducted from November 1, 2022 to November 30, 2023 at a public district hospital, Vietnam. The data collection time was from May 15, 2023 to June 30, 2023 using the SERVPERF (Service Performance) tool for quality assessment.

### 2.3 Study Participants

The study population consisted of outpatients utilizing services at the district hospital. To ensure the study captured the perceptions of typical, non‐expert patients, the inclusion criteria were defined as: (i) aged over 18, (ii) absence of mental health problems, and (iii) not currently employed as health workers at this facility. This exclusion was implemented to avoid potential professional bias, ensuring that the findings accurately reflect the service quality perceptions of the general public rather than those of individuals with “insider” knowledge of hospital operations.

### 2.4 Sample Size Estimation

The required sample size was calculated using a conservative estimate of standard deviation (SD approximately 0.8) based on prior SERVPERF studies. Given the lack of localized pilot data at the time of study design, this provided a baseline to achieve a margin of error of 5%. Post‐hoc assessment of our data (*n* = 190) indicates that our sample size was sufficient to detect associations in our exploratory multiple logistic regression model. And based on a comparable hospital in the province where 67.6 of patients expressed satisfaction with the quality of healthcare services [13], and with a confidence level of 95% and a margin of error of 5%, the sample size required for this study was determined to be 190 outpatient clients. And they were recruited by the convenient sampling with 10 clients per day to mitigate time‐of‐day bias and minimize disruption to clinical operations, the daily recruitment target of 10 participants was systematically distributed across both morning and afternoon shifts.

### 2.5 Research Variables

The independent variables encompassed socio‐demographic factors such as sex, age, education level, occupation, income level, and outpatient visits, while the dependent variable was the patients' perception about healthcare service quality (PHSQ) as in the questionnaire tool.

### 2.6 Research Instrument and Data Collection

The outpatient service quality was assessed using the SERVPERF scale, originally developed by Cronin and Taylor [1, 19]. To ensure the instrument was appropriate for the Vietnamese healthcare context and the post‐pandemic recovery phase, we adapted the scale based on the previous applications by Rasyida et al. [2]. The adaptation process followed a three‐step procedure:

1. Content Validity: The adapted items were reviewed by a panel of three healthcare administrators and two academic experts in public health to ensure the items were relevant to the current post‐pandemic hospital environment.
2. Linguistic Adaptation: The items were translated into Vietnamese and back‐translated to ensure conceptual equivalence.
3. Pilot Testing: A pilot study was conducted with 10 outpatients to evaluate the clarity and reliability of the questions. Feedback from the pilot study led to minor adjustments in the phrasing of items within the “Tangibles” and “Responsiveness” dimensions to improve clarity.

Five study staff (nurses) were trained in 2 days of study protocol and they were responsible for data collection with the 20‐min self‐reported questionnaire in a private setting to ensure the confidentiality, and asked for the written informant consents were obtained from each correspondent prior to data collection.

### 2.7 Data Analysis

Data were analyzed using SPSS 26.0. Descriptive statistics were used to summarize participant characteristics, with continuous variables reported as mean ± standard deviation and categorical variables as frequencies and percentages.

Model fit was evaluated using the following indices: the ratio of chi‐square to degrees of freedom (Χ^2^/df), the comparative fit index (CFI), the Tucker‐Lewis index (TLI), and the root mean square error of approximation (RMSEA). Adequate model fit was determined based on the following established thresholds: Χ^2^/df < 3.0 [20], CFI and TLI values ≥ 0.90 [21], and RMSEA values ≤ 0.08 [21].

The perception of healthcare service quality (PHSQ) is indeed calculated using SERVPERF average scores, with specific cut‐off points to determine low and high perceptions of health service quality. The cut‐off scores for the PHSQ were determined by applying the calculation method of Serkan Narli [22]. The distance value was calculated using the formula: Distance value = (Max − Min)/*n*, whereas, in this case, with a range of 1 to 5, the distance value was (5 − 1)/5 = 0.8. This value was then divided into 5 intervals, each of which has a value of 0.8 units. Therefore, the cut‐off scores for the PHSQ were 4.0 determined as “Good” based on these intervals to categorize the responses from worse to excellent as 1 to 5 [22].

We performed bivariate analysis (chi‐square test) and exploratory multiple logistic regression to identify factors associated with patient perception of healthcare service quality (PHSQ), dichotomized as “low” (< 4.0) versus “high” (≥ 4.0). To facilitate regression analysis, continuous independent variables (Age and Income) were dichotomized into binary categories. The cut‐off points were determined based on the sample distribution and socio‐economic benchmarks: (i) Age: The sample was dichotomized at 45 years old. This cut‐off was selected based on the median age of the respondents, which allows for a balanced comparison between younger adults and middle‐to‐older aged patients who are more likely to utilize chronic care services; (ii) Income: Monthly income was dichotomized $160 based on the median monthly household income. This classification serves to distinguish between low‐income patients, who may face greater financial barriers to healthcare access, and those with higher disposable income, providing a clearer view of the impact of socioeconomic status on service quality perceptions. By utilizing these thresholds, we ensured sufficient statistical power for the logistic regression models while maintaining interpretability of the results.

All statistical tests were two‐sided, with a significance level (α) set at 0.05. Odds ratios (OR) and 95% confidence intervals (95% CI) were reported for regression results. All analyses were conducted to explore associations; hence, these results are considered exploratory rather than confirmatory.

### 2.8 Ethical Consideration

The study was approved by the Ethics Committee of Hanoi University of Public Health, Vietnam, with Decision No. 218/2023/YTCC‐HD3 dated May 4, 2023, and all study participants provided written informed consents.

## 3 Results

### 3.1 Socio‐Demographic Characteristics

A total of 190 out‐patients were successfully enrolled in the study, meeting the specified sample size requirement of 190. Among the participants, 58.9% (*n* = 112) were female, while the proportion of male participants was lower. The age group of 18–44 years accounted for a higher proportion of respondents at 57.9% (*n* = 110) compared to the age group of 45 and above, which made up 42.1% (*n* = 80) of the cohort. Individuals with a high school education level or below represented the highest proportion at 83.7% (*n* = 159). A significant portion of the cohort, 53.7% (*n* = 102) reported occupations as workers and peasants. Furthermore, the proportion of respondents with a monthly income of approximately $160 and below was comparable to those earning $160 and above. Additionally, the sample predominantly comprised participants who had visited the hospital twice or more, accounting for 95.3% (*n* = 181) of the cohort (Table 1).

**Table 1:** Outpatients' characteristics (*n* = 190).

Variables		*N*	Frequency
Sex	Male	78	41.1
	Female	112	58.9
Age group	18–44	110	57.9
	≥ 45	80	42.1
Education	High school and below	159	83.7
	College and above	31	16.3
Occupation	Worker and peasant	102	53.7
	Governmental staff	88	46.3
Monthly income	< $160	96	50.5
	≥ $160	94	49.5
Number of visit	1	9	4.7
	≥ 2	181	95.3

#### 3.1.1 Healthcare Service Quality (HSQ) Score

The reliability of SERVPERF total instrument was good with Cronbach's alpha of 0.96. The reliability of all factors was relatively good with Cronbach's alpha of the factors such as Tangible, Reliability, Responsiveness, Assurance, and Empathy factor of 0.79, 0.87, 0.87, 0.83, and 0.92 respectively.

To address the domain‐specific nuances of service quality, we analyzed the SERVPERF dimensions within our dataset. Among the five dimensions, Empathy (3.99 ± 0.64) and Reliability (4.02 ± 0.64) recorded the lowest mean scores. This internal trend suggests that the post‐pandemic recovery phase continues to challenge the interpersonal and operational aspects of care; the lower empathy scores may reflect the increased administrative burden and patient volumes which limit the time available for individualized patient attention. Conversely, Tangibles (4.28 ± 0.82) and Assurance (4.22 ± 0.73) remained the most resilient dimensions. This highlights that while the “human” and “process” elements of service delivery remain under strain, the physical infrastructure and the professional confidence inspired by clinical staff have remained strong, reflecting the facility's commitment to maintaining a professional and reliable environment during the recovery phase (Table 2).

**Table 2:** Health service quality (HSQ) mean scores by dimensions and perception of healthcare service quality (PHSQ), *n* = 190.

HSQ dimension	Mean score ± SD	Min–Max	Not good PHSQ (< 4.0)	Good PHSQ (≥ 4.0)
Tangible	4.28 ± 0.82	2–5	28 (14.7%)	162 (85.3%)
Reliability	4.02 ± 0.64	2–5	21 (11.1%)	169 (88.9%)
Responsiveness	4.15 ± 0.72	2–5	25 (13.2%)	165 (86.8%)
Assurance	4.22 ± 0.73	2–5	30 (15.8%)	160 (84.2%)
Empathy	3.99 ± 0.64	2–5	28 (14.7%)	162 (85.3%)
PHSQ index	4.13 ± 0.61		47 (24.7%)	143 (75.3%)

**Figure 1:**
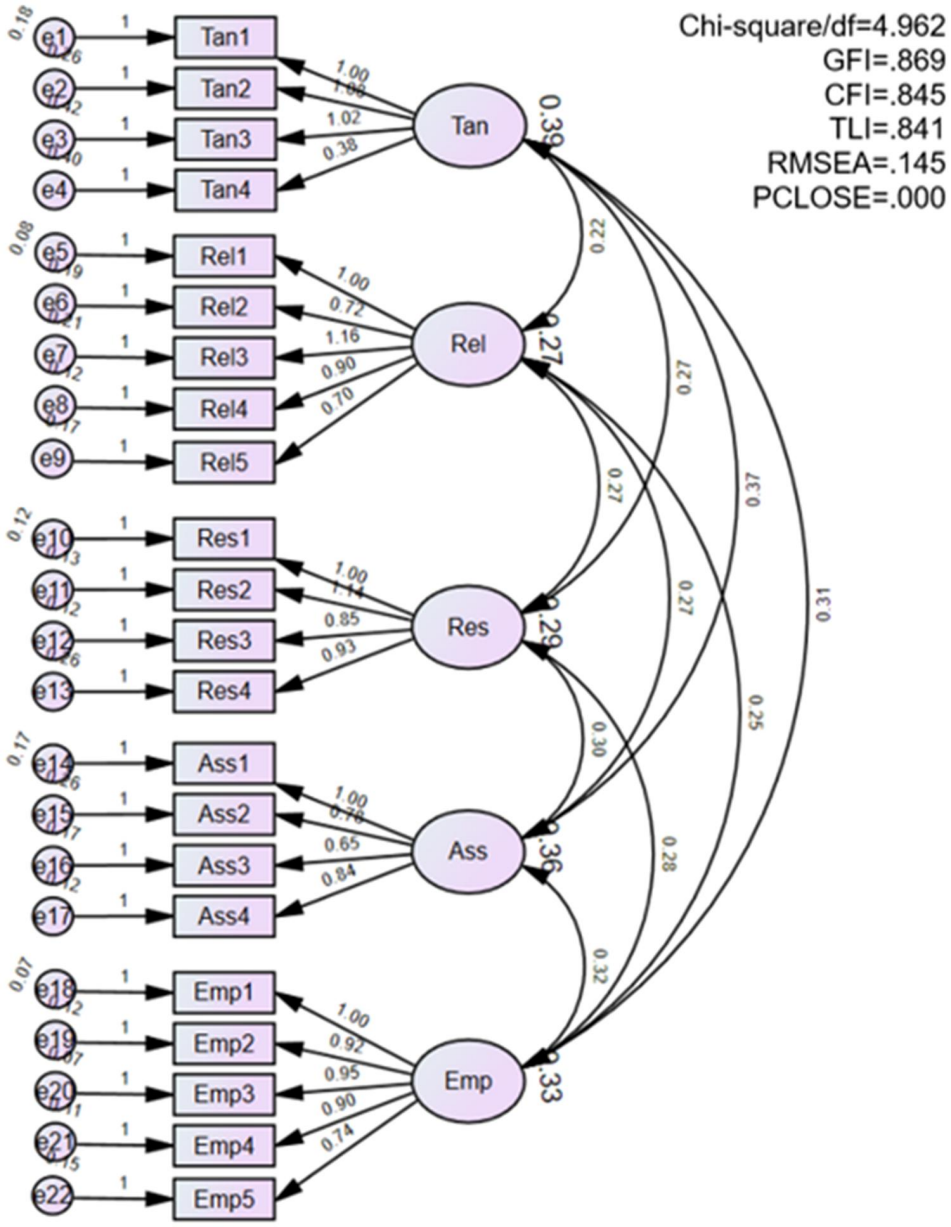
Confirmatory factor analysis (CFA) model for perceived healthcare service quality (PHSQ) constructs. (Tag, Tangible; Rel, Reliability; Res, Responsiveness; Ass, Assurance; Emp, Empathy.)

The CFA was done on the modified tool using Structural Equation Modeling to finalize the PHSQ items. This analysis used survey data from outpatients to confirm the adapted factors identified in the EFA and to validate these constructs. The adjusted PHSQ constructs showed acceptable fit indices, with a chi‐square to degrees of freedom ratio of 4.96 (< 5), and satisfactory values for the root mean square error of approximation (RMSEA) at 0.1, and both the comparative fit index (CFI) and Tucker‐Lewis index (TLI) at 0.845 and 0.841 respectively. As a result, all variables were considered to be significant indicators and predictors of the underlying factors (Figure 1).

In the bivariate analysis, three independent variables—specifically, age group, monthly income, and education were significantly associated with patients' PHSQ. Upon conducting regression analysis, the significance of the results was unchanged though the values of OR and 95% CI had little change. The multiple logistic regression analysis yielded more precise findings. Patients within the age group of 18–44 were observed to have a significantly better PHSQ, being 19.8 times more likely than those aged 45 years and above (OR = 19.8; 95% CI: 6.1–64.3). Furthermore, individuals with monthly income below $160 exhibited a significantly better PHSQ, being 16.2 times more likely than those earning $160 and above (OR = 16.2, 95% CI: 4.5–58.5). Additionally, respondents with a high school education level or below were found to perceive a significantly better PHSQ, being 8.8 times more likely than those with education beyond high school (OR = 8.8, 95% CI: 2.7–28.1) (Table 3).

**Table 3:** Patient's factors related to PHSQ level (*n* = 190).

Characteristic factors		Good PHSQ		Not good PHSQ		Crude OR (95% CI)	Adjusted OR (95% CI)	*p*‐value
		*n*	%	*n*	%			
Sex	Male	61	78.2	17	21.8	1.31 (0.7–2.7)	1.8 (0.7–5.2)	0.43
	Female	82	73.2	30	26.8		1	
Age group	18 – 44	104	94.5	6	5.5	18.2 (7.2–46.3)	19.8 (6.1–64.3)	< 0.01
	≥ 45	39	48.8	41	51.2		1	
Monthly income	< $160	91	94.8	5	5.2	14.7 (5.5–39.5)	16.2 (4.5–58.5)	< 0.01
	≥ $160	52	55.3	42	44.7		1	
Education	≤ High school	134	84.3	25	15.7	13.1 (5.4–31.8)	8.8 (2.7–28.1)	< 0.01
	> High school	9	29.0	22	71.0		1	
Occupation	Worker/Farmer	80	78.4	22	21.6	1.4 (0.7–2.8)	0.9 (0.3–2.6)	0.28
	Others	63	71.6	25	28.4		1	
Number of visit	1	5	55.6	4	44.4	0.4 (0.1–1.5)	0.3 (0.1–2.0)	0.15
	≥ 2	138	76.2	43	23.8		1	

## 4 Discussion

Our research collected feedback on healthcare service quality from a sample of 190 outpatient clients. Data validation was conducted using Cronbach's alpha coefficient, demonstrating high reliability (α = 0.96). The average SERVPERF score was 4.13. Using the PHSQ index with a cutoff of 4.0, our study determined a prevalence of 75.3% (*n* = 143) as having “good” PHSQ. Patient characteristics such as age, monthly income, and education were significantly associated with PHSQ levels, with odds ratios of 19.8, 16.2, and 8.8, respectively.

When comparing our mean SERVPERF score of 4.13 to historical benchmarks, we observe a nuanced trend. Our findings indicate a slight decline compared to the pre‐pandemic score of 4.37 [16] and the pandemic‐era scores of 4.23 and 4.33 [17, 18]. This suggests that the “pandemic‐response unity”—often characterized by heightened patient appreciation for healthcare providers—may be dissipating as the health system returns to routine operations. However, our score of 4.13 is notably higher than the 3.78 reported in recent post‐pandemic assessments [23]. While variations in scores across these studies may be attributed to differences in clinical settings, geographical catchment areas, and patient demographics, our facility demonstrates greater resilience in service delivery. This resilience is likely rooted in the administrative agility implemented during the recovery phase (Table 4).

**Table 4:** Comparative analysis of SERVPERF mean scores across pandemic and post‐pandemic periods.

Period	Benchmark PHSQ score	Source
Pre‐COVID‐19	4.37	Hien HT et al. [16]
During COVID‐19 (2020)	4.23	Hai LTT et al. [6]
During COVID‐19 (2021)	4.33	Thanh TQ et al. [18]
Our study (Post‐COVID)	4.13	This Study
Post‐COVID (2023)	3.78	Chung NTK et al. [23]

While the quantitative SERVPERF scores indicate a relative stability in service quality, qualitative insights from patients and healthcare staff suggest a perceived decline in care delivery. Global evidence consistently demonstrates that healthcare professionals perceived a decline in healthcare quality following the COVID‐19 pandemic. A large international survey involving 2707 healthcare workers across 60 countries reported that over half experienced burnout, which was strongly associated with increased workload and critical decision‐making under pressure [24]. Similarly, multi‐country physician surveys indicated that more than half of doctors reported increased stress and workload, with many acknowledging that burnout adversely affected the quality of care delivered [25]. These findings are further supported by systematic reviews confirming that physician burnout has become a global phenomenon with significant implications for patient safety and healthcare quality [26].

The assessment of service quality has increasingly relied on patient perceptions. Using the PHSQ index (cutoff 4.0), we observed that despite the SERVPERF score of 4.13, the proportion of “good” PHSQ was 75.3%. The variability in SERVPERF scores (4.13 ± 0.61) is reflected by a recorded range of 3.52–4.74. This discrepancy is statistically consistent with the standard deviation exceeding 0.5; the marked prevalence of SERVPERF scores clustering below 4.0 significantly influences the proportion of “good” PHSQ.

The PHSQ index facilitated the precise assessment of patients' PHSQ and was utilized to ascertain the impact of associated factors on their perceptions using dichotomous values. In our bivariate analysis, it was observed that four independent variables—namely, age group, monthly income, education, and occupation—exhibited significant associations with patients' PHSQ. However, subsequent regression analysis yielded noteworthy changes in the significance of these findings. While age group, monthly income, and education continued to demonstrate significant associations with patients' PHSQ, the association between occupation and patients' PHSQ was no longer evident. This is understandable that in bivariate analysis, the relationship between two variables is examined without considering other potential influencing factors. However, in regression analysis, multiple variables can be included simultaneously to assess their individual effects on the dependent variable while controlling for other relevant factors. By reducing the number of associated factors from bivariate analysis to regression analysis, researchers can identify the factors that have more substantial associations with the dependent variable, as the effects of confounding variables are controlled or adjusted for in the regression model [27]. Prior research conducted in Vietnam, both before and during the COVID‐19 pandemic, primarily focused on examining healthcare service quality scores through the lens of healthcare supplier‐related factors [8]. These studies identified several influential elements including human resources, which manifested in the favorable evaluation of staff communication and behavioral competencies, as well as the organization of medical examination and treatment processes. Additionally, positive ratings from patients were associated with robust physical infrastructure factors, while medical information was identified as an area requiring improvement in order to enhance service quality. Furthermore, the presence of an adequate number of personnel, structured training processes for healthcare staff, well‐equipped facilities, and streamlined medical examination and treatment procedures were identified as positive drivers of healthcare service quality. In contrast, cramped examination room space and unclear signage were recognized as adverse factors negatively impacting service quality [23].

Notably, these previous studies did not leverage the PHSQ index to explore patient‐related factors influencing their perceptions. While studies in other countries have examined patient‐related factors, the association between these factors and healthcare service quality varies according to health settings, countries, and cultures. For instance, a study conducted in Sweden in 2007 pertaining to emergency care found that older patients, particularly those with excellent health status, demonstrated the highest satisfaction levels, with 90% expressing contentment, while younger patients exhibited notably lower satisfaction, with only 54% reporting a positive experience. Additionally, individuals with lower levels of education exhibited greater satisfaction in comparison to their counterparts with higher educational attainment [28].

In our study, patients aged between 18 and 44 years were noted to exhibit a markedly better PHSQ, showcasing a likelihood 19.8 times higher than their counterparts aged 45 years and older (OR = 19.8; 95% CI: 6.1–64.3). The implication of a better PHSQ in this context is that younger patients may have had a better understanding of post‐pandemic healthcare processes and protocols, leading to heightened satisfaction with their healthcare experiences. Further exploration of this association can provide valuable insights for tailoring healthcare communication and services to different age groups.

Moreover, patients with a monthly income below $160 demonstrated a substantially better PHSQ, with a likelihood 16.2 times greater than individuals earning $160 and above (OR = 16.2; 95% CI: 4.5–58.5). The relationship between income and expectations regarding healthcare services is complex and can be influenced by various factors. While it is not accurate to simply generalize that individuals with higher incomes have higher expectations regarding their care services, it is possible that they may have different perceptions of the current health service based on their experiences and access to resources. This finding indicates that there may be disparities in how individuals from different income brackets perceive the quality of healthcare services. It is important to consider that individuals with higher incomes may indeed have higher expectations of healthcare services due to their ability to access a wider range of healthcare options and may have different standards based on their experiences. However, our study highlights that individuals with lower incomes demonstrated better PHSQ, suggesting that they may place a higher value on the care they receive relative to their income level. The influence of socioeconomic status—specifically income—on service expectations is well‐documented in health services literature. Our findings align with the “Consumerist Expectation” model, which suggests that individuals with higher income and socioeconomic standing often possess higher baseline expectations regarding “Reliability” and “Assurance” [29]. This is theorized to stem from a greater sense of “customer agency,” where patients who are more affluent perceive healthcare as a commodified service, leading them to demand higher standards of efficiency and personalization [30]. Conversely, literature also highlights an “Adaptive Expectation” phenomenon among lower‐income populations, where patients may report high satisfaction scores despite objective gaps in quality [30]. This is often attributed to a “pragmatic adaptation” where patients with limited financial resources prioritize basic access and survival over luxury dimensions of service, effectively recalibrating their expectations to match the realities of public facility limitations [5, 31],

A similar trend was identified concerning the education factor, wherein respondents with a high school education level or below perceived a notably higher PHSQ, being 8.8 times more likely than those with educational attainment beyond high school (OR = 8.8; 95% CI: 2.7–28.1).

Considering our findings, the intricacies involved in enhancing healthcare service quality in Vietnam and comparable low‐income nations in the post COVID‐19 pandemic call for holistic measures that take into account the influence of economic obstacles, and resource constraints on service excellence. This approach may entail specific interventions aimed at (i) bolstering service capabilities, (ii) mitigating financial pressures, (iii) upgrading healthcare infrastructures, and (iv) fostering the professional growth of healthcare practitioners. Our suggestions are quite a study in Thailand that suggested six priority areas having challenges in the healthcare system to improve healthcare service quality including: (i) human resources, (ii) environment, (iii) communication, (iv) local governance, (v) social determinants of health, and (vi) medical technology [23]. Furthermore, our study findings suggest that we should also encompass a thorough examination of the subtle effects that patient demographic variables which are sensitive to pandemic crises have on the perception of healthcare service quality.

While this cross‐sectional, single‐site study provides critical insights into the post‐pandemic recovery of outpatient services, it is subject to certain limitations that offer a roadmap for future research. First, the cross‐sectional design limits our ability to establish causality or track the trajectory of patient perceptions over time. The inclusion of six predictor variables for a sample of *N* = 190, while statistically viable for an exploratory model, approaches the lower boundary of the recommended “Events Per Predictor” (EPP) rule‐of‐thumb. Consequently, there is a risk of overfitting, and the stability of the regression estimates should be interpreted with caution. This is particularly evident in the “single outpatient visit” subgroup (*N* = 9), where the wide confidence interval (0.1–2.0) indicates limited statistical precision for this specific estimate. As such, these findings should be viewed as exploratory, and we recommend that future larger‐scale, multi‐center studies be conducted to validate these associations with greater statistical power. Finally, to mitigate the potential biases inherent in self‐reported quantitative surveys, future investigations should employ a mixed‐methods approach, combining SERVPERF data with in‐depth qualitative interviews with both patients and healthcare providers to uncover the “why” behind the quantitative scores.

## 5 Conclusion

This study provides a post‐pandemic evaluation of PHSQ, identifying high performance scores yet a lower prevalence of “good” perception compared to previous benchmarks. Younger age, lower income, and lower education level were identified as key predictors of higher PHSQ. These results suggest that hospital management should transition from passive monitoring to active quality intervention. Our recommendations focus on Reliability through digital process standardization and Empathy through workflow optimization. By automating administrative workflows (to improve Reliability) and investing in communication training (to improve Empathy), the facility can effectively target the specific areas of service decline identified in this study.

## Author Contributions

**Nguyen Duc Thanh:** conceptualization, methodology, data curation, writing – original draft. **Tran Chi Thanh:** methodology, writing – review and editing. **Ha Thi Minh Nguyet:** conceptualization, methodology, data curation, writing – review and editing. **Chu Huyen Xiem:** conceptualization, data curation.

## Funding

The authors have nothing to report.

## Conflicts of Interest

The authors declare no conflicts of interest.

## Declaration of AI Use

We did not use AI tools for generating our texts, just for grammatical suggestions.

## Data Availability

The data that support the findings of this study are available from the corresponding author upon reasonable request. The data sets used and/or analyzed during the current study available from the corresponding author on reasonable request.
